# Ovariectomy Enhances Carcass Performance and Meat Quality by Modulating Muscle Development and Lipid Metabolism in Wuding Hens

**DOI:** 10.3390/ani15213183

**Published:** 2025-10-31

**Authors:** Le Zhang, Xiaoqi Xu, Wenbin Dao, Yongwang Miao

**Affiliations:** 1 College of Animal Science and Technology, Yunnan Agricultural University, Kunming 650201, China; 2 Institute of Animal Genetics and Breeding, Yunnan Agricultural University, Kunming 650201, China

**Keywords:** Wuding hens, ovariectomy, muscle development, meat quality, lipid metabolism

## Abstract

**Simple Summary:**

Estrogen is an important hormone that shapes muscle growth and function, but its role in female chickens has not been fully clarified. To address this, we used Wuding hens, a traditional Chinese breed, and surgically removed the ovaries to establish an estrogen-deficient model. Growth performance, muscle quality, and lipid metabolism were then compared with intact hens that retained normal estrogen production. By the end of the growth cycle, the ovariectomized hens showed greater body weight and higher carcass yield than controls. Their leg muscles were also more tender, even though the morphology of muscle fibers did not differ between groups. Lipid analysis revealed that intramuscular triglycerides increased at specific growth stages, pointing to altered fat deposition. At the molecular level, transcriptomic and proteomic profiling indicated that estrogen influences both muscle development and energy metabolism through changes in signaling pathways and gene expression. Taken together, these findings demonstrate that estrogen has a complex role in regulating muscle traits in hens. Beyond advancing our understanding of hormone action in poultry, this work also provides insights that may be useful for improving meat quality in female chickens.

**Abstract:**

Estrogen is a key regulator of skeletal muscle growth and metabolism in birds, yet its specific roles in female chickens remain poorly defined. To address this gap, we established an estrogen-deficient model by surgically removing the ovaries of Wuding hens, a Chinese indigenous slow-growing breed. Growth traits, carcass yield, and meat quality were evaluated across different ages, complemented by histological examination, serum biochemical analysis, and multi-omics approaches (transcriptomics, proteomics, and lipidomics). Ovariectomized hens maintained somatic growth for a longer period and reached greater body weight and carcass yield at 330 days compared with intact controls. Thigh muscle tenderness was also enhanced in the absence of estrogen, despite no long-term differences in muscle fiber morphology. Lipidomic analysis revealed a transient increase in intramuscular triglyceride content at mid-growth (240 days), pointing to altered lipid storage and distribution. Integrated omics profiling further demonstrated significant changes in the mitogen-activated protein kinase (MAPK) and mechanistic target of rapamycin (mTOR) signaling pathways, accompanied by differential expression of key metabolic and structural genes, including mitogen-activated protein kinase 8 (*MAPK8*), fatty acid binding protein 4 (*FABP4*), ankyrin 1 (*ANK1*), and coenzyme Q6 monooxygenase (*COQ6*). These molecular adjustments suggest that estrogen withdrawal triggers broad reprogramming of muscle signaling and lipid metabolism. Overall, this study highlights the multifaceted role of estrogen in coordinating growth, muscle quality, and lipid homeostasis in hens and provides a functional model for studying estrogen deficiency in poultry with implications for meat quality improvement.

## 1. Introduction

Skeletal muscle growth and meat quality in poultry are shaped by a combination of genetic background, nutrition, and endocrine regulation. Among these factors, estrogen has a central role in muscle development, metabolism, and tissue differentiation. While its function in reproduction is well established, how estrogen regulates muscle biology and meat quality in female chickens is still poorly understood [1]. Most previous work has concentrated on males or has emphasized hormone-independent pathways, leaving the influence of estrogen in hens largely unexplored.

A practical approach to study estrogen function in vivo is to induce estrogen deficiency. In chickens, ovariectomy—the surgical removal of the ovaries—eliminates the major source of circulating estrogen and leads to a sharp decline in estradiol levels [2]. This intervention creates a physiologically relevant model for evaluating how the absence of estrogen affects somatic growth and metabolism. Comparisons between ovariectomized hens and intact controls can therefore clarify estrogen’s role in muscle development, energy balance, and meat quality traits, extending our view of its actions beyond reproduction.

In poultry science, most endocrine studies have focused on males. Caponization in roosters is a well-documented example, producing heavier carcasses, greater fat deposition, and meat with higher tenderness and flavor compared to intact males [3]. These outcomes have made male caponization both a production strategy and a biological model. In contrast, very few studies have assessed the consequences of ovariectomy in hens. Early findings suggest that estrogen removal may delay sexual maturity, extend growth duration, and improve tenderness, but often with increased fat deposition and reduced feed efficiency [4]. The mechanisms underlying these changes, however, remain largely unknown.

Beyond poultry, research in mammals provides evidence that estrogen deficiency profoundly influences muscle structure and metabolism. For example, ovariectomy in rodents and postmenopausal models in humans are associated with reduced muscle mass, altered mitochondrial function, and increased lipid accumulation [5,6,7]. These studies highlight the systemic role of estrogen in coordinating energy homeostasis and skeletal muscle plasticity, but comparable data in chickens are scarce. Moreover, while several studies have linked estrogen to lipid metabolism and intramuscular fat deposition in cattle and pigs [8,9], the specific molecular pathways in poultry remain poorly characterized. These methodological gaps justify the need for a comprehensive investigation of estrogen’s role in female chickens.

The Wuding chicken, a slow-growing indigenous breed from Yunnan Province, pro-vides a valuable genetic background for such work. Known for its distinctive meat and egg quality, it has undergone limited artificial selection, which preserves many of its native physiological and metabolic characteristics [10,11]. These features make Wuding chickens a suitable model for testing hormonal interventions like ovariectomy, particularly for understanding growth and meat quality in non-commercial breeds.

In this study, we established an estrogen-deficient model by surgically ovariectomizing Wuding hens and assessed its effects across different growth stages. We combined carcass and meat quality measurements with histological analysis, transcriptomic and proteomic profiling, and targeted lipidomics. Based on prior findings, we hypothesized that estrogen deficiency would prolong growth and alter muscle quality in hens by remodeling lipid metabolism and key signaling pathways. By integrating multi-omics approaches, this work aims to clarify the mechanisms by which estrogen regulates muscle development and metabolism, thereby filling an important knowledge gap in poultry biology.

## 2. Materials and Methods

### 2.1. Experimental Animals

All experimental birds were obtained from the breeding facility of Shouyu Agricul-ture and Technology Development Co., Ltd. (Wuding, China). Thirty-six healthy Wuding hens, originating from the same hatch and raised under uniform feeding and management conditions, were selected. The population included both intact hens and ovariectomized hens, with surgery carried out at 50 days of age. At each sampling point, birds were randomly chosen from the experimental groups: 12 hens at 160 days, 12 hens at 240 days, and 12 hens at 330 days. All birds were reared under the same housing conditions, received diets formulated to meet NRC nutritional standards, and were managed following standard poultry husbandry practices.

All birds were reared in wire cages, with two hens housed per cage to allow social interaction while preventing overcrowding. The poultry house was maintained under controlled environmental conditions, including temperature, humidity, and light cycles, according to local poultry husbandry standards. Birds had free access to water supplied through nipple drinkers and were fed a standard commercial corn–soybean basal diet formulated to meet NRC (1994) nutritional requirements for growing chickens. Feed and water were replenished daily, and the health and welfare of the birds were checked twice per day throughout the experimental period.

### 2.2. Ovariectomy Procedure

Ovariectomy was performed at 50 days of age using a modified lateral laparotomy approach designed to minimize tissue damage and reduce postoperative complications. Birds were restrained on their left side at a 45° angle on the surgical platform. Feathers at the surgical site were plucked, and the skin was disinfected with povidoneiodine; surrounding feathers were moistened to improve visibility. A 2–3 cm longitudinal incision, approximately 0.5 cm deep and parallel to the ribs, was made at the lateral flank, which is smaller and less invasive than the conventional ventral approach. The incision was gently widened with retractors, and the peritoneal and air sac membranes were pierced with the tip of a surgical spoon. The ovarian capsule was opened and the ovary was carefully detached from its attachment using blunt dissection. The ovary was ligated at the ovarian pedicle with sterile silk suture and excised. To facilitate drainage and reduce the risk of infection, ~0.1 cm of skin at one end of the incision was trimmed. Topical antibiotics were applied immediately after surgery as part of postoperative care, avoiding the need for systemic treatment.

For the sham-operated (control) group, hens underwent the same procedures, including anesthesia, feather removal, skin disinfection, incision, and gentle peritoneal entry. However, their ovaries were left intact and no excision was performed. The incision was closed after visual confirmation of ovarian integrity, and the same topical antibiotic treatment was applied.

### 2.3. Sample Collection and Processing

At each designated growth stage (160, 240, and 330 days), a total of 12 hens (6 ovariectomized and 6 intact controls) were euthanized, and samples were collected immediately postmortem. Leg muscle (gastrocnemius) and breast muscle (pectoralis major) were harvested for meat quality evaluation, histological examination, transcriptomic and proteomic analysis, and lipidomics. In addition, blood was collected via venipuncture, and serum was isolated by centrifugation using an Eppendorf 5810R centrifuge (Eppendorf AG, Hamburg, Germany) at 3000 rpm for 10 min. All tissue and serum samples were snap-frozen in liquid nitrogen and stored at −80 °C until further use.

### 2.4. Measurement of Growth and Carcass Traits

Growth performance and carcass traits were evaluated at 160, 240, and 330 days of age. Four standardized indices were recorded to characterize carcass development. Live body weight (LBW) was measured immediately prior to slaughter following a 12 h feed withdrawal, while water was freely available. Post-bleeding weight (PBW) was determined after complete exsanguination by severing the jugular vein, with feathers, viscera, head, and legs intact. Post-bleeding and defeathering weight (PBDW) was recorded after feather removal, with viscera, head, and legs still present. Finally, eviscerated carcass weight (ECW) was obtained after removal of viscera, head, and legs at the hock joint, representing the edible portion of the carcass.

All weights were determined immediately after processing using a calibrated electronic balance (Model FA2204B, Shanghai Precision Scientific Instrument Co., Shanghai, China; accuracy ±0.01 g). The balance was verified daily using standard reference weights to minimize measurement error and ensure consistency across sampling stages.

### 2.5. Meat Quality Trait Assessment

Samples of both leg (gastrocnemius) and breast (pectoralis major) muscles were collected and analyzed for muscle mass (g), cooking loss (%), water-holding capacity at 45 min and 24 h postmortem (%), Warner–Bratzler shear force (N), and colorimetric parameters (lightness L*, redness a*, and yellowness b*). Muscle mass was determined using an electronic balance (ME204E, Mettler-Toledo, Greifensee, Switzerland; accuracy ±0.001 g). Cooking loss was measured by recording the weight of standardized muscle samples (approximately 30 g) before and after cooking in sealed plastic bags immersed in a water bath at 80 °C for 30 min, followed by cooling to room temperature and reweighing. Cooking loss was calculated as the percentage difference between pre- and post-cooking weights.

Muscle color parameters (L*, a*, b*) were assessed using a meat colorimeter (CR-400, Konica Minolta, Tokyo, Japan) under standardized room lighting (D65 illuminant, 20 ± 1 °C). Measurements were taken at three random points on the freshly cut surface of each sample, and the average was used for statistical analysis. Water-holding capacity was determined at 45 min and 24 h postmortem by pressing standardized muscle portions (approximately 2 g) between filter papers under a defined load, and the expressed juice area was quantified following the Chinese National Standard “Analytical Methods for Meat and Meat Products”.

Tenderness was evaluated using a Warner–Bratzler shear force instrument (TA.XT Plus, Stable Micro Systems, Surrey, UK). Cooked samples (1 cm × 1 cm × 3 cm) were cut parallel to the muscle fibers, and shear force was measured by cutting perpendicularly to the fibers with a V-shaped blade at a constant crosshead speed of 200 mm/min. For each muscle, three subsamples were tested, and the average value was recorded.

### 2.6. Histological Analysis

Muscle tissue samples were collected from the central region of the gastrocnemius (leg) and pectoralis major (breast) muscles of six hens per group at each sampling point (160, 240, and 330 days of age). Approximately 1 cm^3^ of tissue was excised, fixed in 4% paraformaldehyde for 24 h, dehydrated, and embedded in paraffin. Serial sections were cut at a thickness of 5 μm and stained with hematoxylin and eosin (H&E) following standard protocols. For each bird, three non-overlapping sections were prepared. In each section, five randomly selected fields of view were captured at 200× magnification using a light microscope (Nikon Eclipse Ci-L, Nikon, Tokyo, Japan). Muscle fiber cross-sectional area and fiber density were measured using ImageJ software (version 1.8.0, NIH, Bethesda, MD, USA). The average of all measurements per bird was used for statistical analysis to reduce intra-sample variability.

### 2.7. Serum Biochemical Analysis

Blood samples were collected from the wing vein of each hen and centrifuged at 3000 rpm for 10 min using an Eppendorf 5810R centrifuge (Eppendorf AG, Hamburg, Germany) to obtain serum. Biochemical parameters, including alanine aminotransferase (ALT, Cat. #I123), total protein (TP, Cat. #S0211S), triglycerides (TG, Cat. #S0219S), and creatine kinase (CK, Cat. #T0906), were quantified using commercial kits (Beyotime Biotechnology, Shanghai, China) on a Hitachi 7020 Automatic Biochemical Analyzer (Hitachi High-Technologies Corporation, Tokyo, Japan). All measurements were conducted in triplicate to ensure accuracy and reproducibility.

### 2.8. Transcriptome Sequencing

Total RNA was isolated from leg muscle (gastrocnemius) tissue using TRIzol reagent (Invitrogen, Carlsbad, CA, USA). RNA quality and integrity were evaluated with an Agilent 2100 Bioanalyzer (Agilent Technologies, Santa Clara, CA, USA). Libraries were constructed and sequenced on the Illumina NovaSeq platform (Illumina, San Diego, CA, USA). Differentially expressed genes (DEGs) were defined using |log_2_FC| > 1 and a false discovery rate (FDR) < 0.05. Functional interpretation was carried out through Gene Ontology (GO) annotation and Kyoto Encyclopedia of Genes and Genomes (KEGG) enrichment analyses.

### 2.9. Proteomic Analysis

Proteins were extracted from gastrocnemius muscle tissue, digested with trypsin, and labeled using tandem mass tags (TMT). Peptides were then separated and analyzed using high-resolution liquid chromatography-tandem mass spectrometry (LC-MS/MS). Differentially expressed proteins (DEPs) were defined as those with |log_2_FC| > 1 and FDR < 0.05. Functional annotation and enrichment analyses were conducted using GO and KEGG databases to identify pathways associated with muscle protein dynamics and metabolism.

### 2.10. Lipid Accumulation and Lipidomics Analysis

Intramuscular fat deposition was initially assessed histologically in gastrocnemius samples. For lipidomics, lipids were extracted using a modified Bligh and Dyer method [12]. Samples were analyzed with ultra-high-performance liquid chromatography coupled to quadrupole time-of-flight mass spectrometry (UHPLC-QTOF-MS). Lipid species were identified and quantified using LipidSearch software (version 5.1, Thermo Fisher Scientific, Waltham, MA, USA). Changes in lipid class composition and enrichment of metabolic pathways were compared across growth stages and between treatments.

### 2.11. Statistical Analysis

All statistical analyses were conducted using SPSS software (version 25.0; IBM Corp., Armonk, NY, USA). Data were analyzed separately for each treatment and age group. Comparisons between ovariectomized and intact hens at the same age were performed using independent-sample *t*-tests. Differences among ages within the same treatment group were evaluated using one-way analysis of variance (ANOVA) followed by Tukey’s post hoc test. A *p* < 0.05 was considered statistically significant, and *p* < 0.01 was considered highly significant. All data are presented as mean ± SEM. Figures and data visualizations were prepared using GraphPad Prism 8.0 (GraphPad Software, San Diego, CA, USA) and R software (version 4.2.0).

## 3. Results

### 3.1. Effects of Ovariectomy and Growth Duration on Growth and Carcass Traits in Wuding Hens

**Figure 1:**
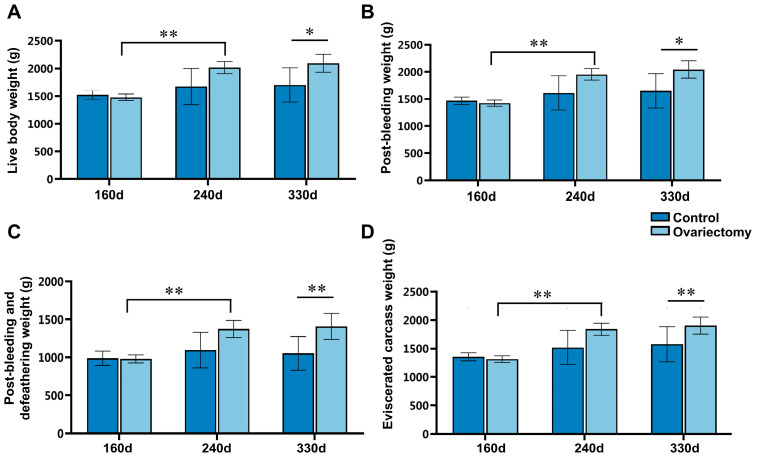
Effects of ovariectomy and growth duration on carcass-related traits in Wuding hens. (**A**) Live body weight (LBW), (**B**) post-bleeding weight (PBW), (**C**) post-bleeding and defeathering weight (PBDW), and (**D**) eviscerated carcass weight (ECW) were measured at 160, 240, and 330 days of age in both ovariectomized and intact hens. Values are presented as mean ± SEM (*n* = 6). * *p* < 0.05, ** *p* < 0.01.

Growth and carcass performance were evaluated in ovariectomized and intact Wuding hens by measuring live body weight (LBW), post-bleeding weight (PBW), post-bleeding and defeathering weight (PBDW), and eviscerated carcass weight (ECW) at 160, 240, and 330 days of age. In ovariectomized hens, all carcass-related indices increased significantly from 160 to 240 days (*p* < 0.01). By 330 days, each parameter was significantly higher than that of age-matched intact hens (*p* < 0.05), indicating that removal of ovarian estrogen extended the growth phase and enhanced carcass yield in the later stages of rearing (Figure 1).

**Figure 2:**
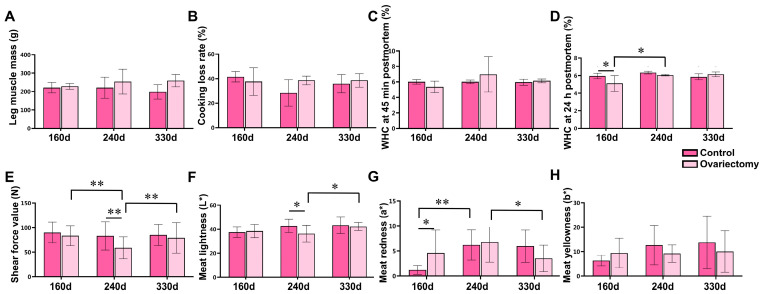
Effects of ovariectomy and age on leg muscle quality traits in Wuding hens. (**A**) Leg muscle mass (g). (**B**) Cooking loss rate (%) of leg muscle. (**C**) Water-holding capacity (WHC) at 45 min postmortem (%). (**D**) Water-holding capacity (WHC) at 24 h postmortem (%). (**E**) Shear force value (N). (**F**) Meat lightness (L*). (**G**) Meat redness (a*). (**H**) Meat yellowness (b*). Values are presented as mean ± SEM (*n* = 6 hens per group). * *p* < 0.05, ** *p* < 0.01.

Meat quality traits were further examined in leg muscles (Figure 2). Neither age nor ovariectomy significantly influenced leg muscle weight, cooking loss, or water-holding capacity (WHC) at 45 min postmortem (*p* > 0.05) (Figure 2A–C). However, at 160 days, the 24 h WHC was greater in intact hens than in ovariectomized hens (*p* < 0.05) (Figure 2D). Notably, tenderness was affected by estrogen removal: at 240 days, shear force values were markedly lower in ovariectomized hens compared with controls and with other time points within the same group (*p* < 0.01), demonstrating enhanced tenderness at this stage (Figure 2E). Muscle color traits were also influenced. Lightness (L*) and redness (a*) varied significantly with both treatment and age (*p* < 0.05) (Figure 2F,G), while yellowness (b*) was not affected (*p* > 0.05) (Figure 2H).

**Figure 3:**
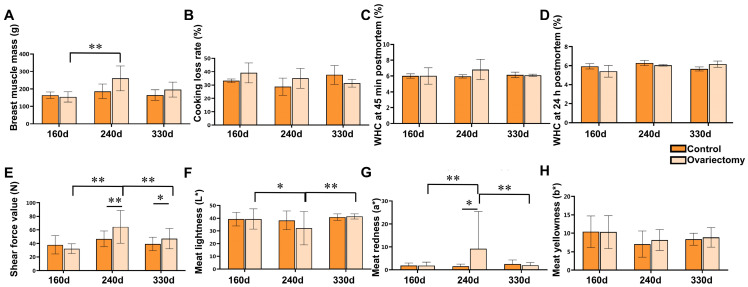
Effects of ovariectomy and age on breast muscle quality traits in Wuding hens. (**A**) Breast muscle mass (g). (**B**) Cooking loss rate (%) of breast muscle. (**C**) Water-holding capacity (WHC) at 45 min postmortem (%). (**D**) Water-holding capacity (WHC) at 24 h postmortem (%). (**E**) Shear force value (N). (**F**) Meat lightness (L*). (**G**) Meat redness (a*). (**H**) Meat yellowness (b*). Values are presented as mean ± SEM (n = 6 hens per group). * *p* < 0.05, ** *p* < 0.01.

In breast muscles (Figure 3), cooking loss and WHC at both 45 min and 24 h postmortem were unaffected by ovariectomy or growth stage (*p* > 0.05) (Figure 3B–D). Breast muscle mass reached its highest value at 240 days, significantly exceeding that at 160 days (*p* < 0.05), followed by a modest decline by 330 days (Figure 3A). Tenderness showed a different pattern from leg muscles: ovariectomized hens exhibited significantly higher shear force at 240 days than controls (*p* < 0.01). Within the ovariectomized group, shear force at 240 days was also greater than at 160 and 330 days (*p* < 0.01) (Figure 3E), suggesting a transient reduction in tenderness during mid-growth. For color parameters, ovariectomized hens at 240 days displayed reduced lightness (L*) and elevated redness (a*) relative to other ages (*p* < 0.01) (Figure 3F,G), changes that may reflect increased myoglobin concentration or muscle fiber maturation.

### 3.2. Effects of Ovariectomy and Growth Period on Muscle Morphology and Serum Biochemical Parameters in Wuding Hens

**Figure 4:**
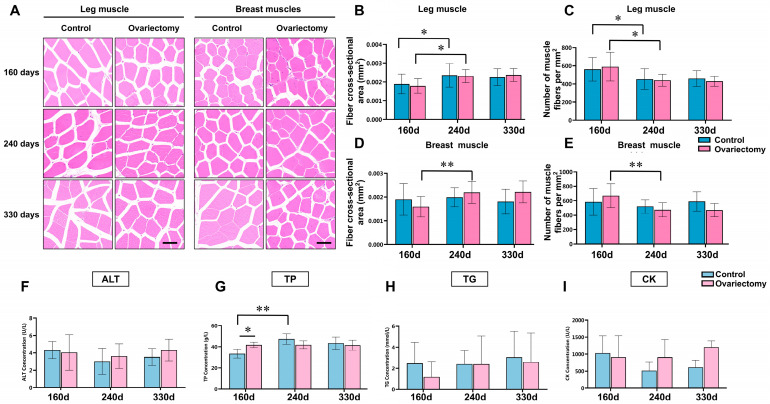
Histological characteristics of skeletal muscle and serum biochemical indices in ovariectomized and control Wuding hens. (**A**) Representative H&E stained sections of gastrocnemius and pectoralis major muscles at three time points. Bar = 100 μm. (**B**,**C**) Quantification of gastrocnemius muscle fiber cross-sectional area (**B**) and fiber density (**C**). (**D**,**E**) Quantification of pectoralis major muscle fiber cross-sectional area (**D**) and fiber density (**E**). (**F**–**I**) Serum biochemical parameters, including alanine aminotransferase (ALT, (**F**)), total protein (TP, (**G**)), triglycerides (TG, (**H**)), and creatine kinase (CK, (**I**)). * *p* < 0.05, ** *p* < 0.01. n = 6.

Hematoxylin–eosin (H&E) staining analysis was conducted to examine structural changes in leg (gastrocnemius) and breast (pectoralis major) muscles of Wuding hens following ovariectomy. Samples were collected at 160, 240, and 330 days of age from both ovariectomized and intact groups (Figure 4A). Muscle fiber cross-sectional area and fiber density were quantified to evaluate age- and treatment-related differences. At each age, no significant differences in either parameter were detected between the ovariectomized and control groups (*p* > 0.05). In both groups, however, clear age-related remodeling was observed: hens at 240 days showed larger fiber areas and reduced fiber densities compared with 160-day-old birds (*p* < 0.05), reflecting muscle fiber hypertrophy and a decline in fiber packing density. Similar changes were also evident within the ovariectomized group between 160 and 240 days (*p* < 0.05) (Figure 4B–E). These results suggest that estrogen deficiency does not alter the general trajectory of age-dependent muscle remodeling, although it may influence the timing or extent of these changes.

To investigate possible systemic effects, serum biochemical indices were analyzed. Alanine aminotransferase (ALT), triglycerides (TG), and creatine kinase (CK) showed no significant differences between groups or among ages (*p* > 0.05) (Figure 4F,H,I), indicating that liver function, lipid metabolism, and muscle damage markers remained stable under the conditions of this study. By contrast, total protein (TP) levels exhibited notable variation. At 160 days, ovariectomized hens had significantly higher TP than their age-matched controls (*p* < 0.05). In intact hens, TP increased significantly from 160 to 240 days (*p* < 0.05) (Figure 4G). These findings suggest that both ovariectomy and age-related maturation may promote systemic protein synthesis or turnover, potentially reflecting shifts in endocrine status or nutrient utilization.

### 3.3. Effects of Ovariectomy on the Transcriptomic and Proteomic Landscape of Skeletal Muscle in Wuding Hens

**Figure 5:**
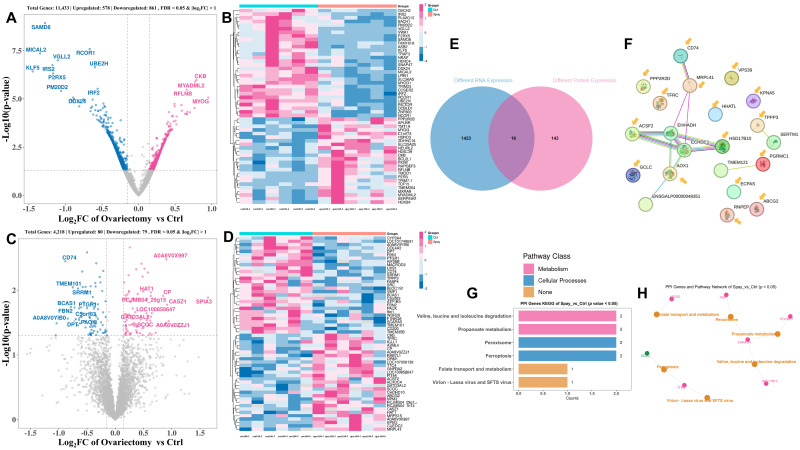
Transcriptomic and proteomic profiling of gastrocnemius muscle in ovariectomized and control Wuding hens at 160 days of age. (**A**,**B**) Differentially expressed genes (DEGs) identified by transcriptomic analysis (**A**) and their normalized expression levels (**B**). (**C**,**D**) Differentially expressed proteins (DEPs) identified by proteomic analysis (**C**) and their relative abundance (**D**). (**E**) Selection of key genes based on cross-omics integration. (**F**) Protein–protein interaction (PPI) network analysis of 16 representative genes. (**G**) Enrichment of signaling pathways associated with key genes. (**H**) Correlation analysis among the enriched pathways.

To investigate how estrogen deficiency influences skeletal muscle at the molecular level, we conducted integrated transcriptomic and proteomic analyses of leg muscle (gastrocnemius) collected from ovariectomized and intact Wuding hens at 160 days of age. RNA sequencing detected 11,433 genes in total, of which 1439 were differentially expressed (DEGs; |log_2_FC| > 1, *FDR* < 0.05). These included 578 genes upregulated and 861 downregulated in ovariectomized hens (Figure 5A). A heatmap of the top 50 DEGs is presented in Figure 5B. Proteomic profiling identified 4218 proteins, with 159 showing significant differences between groups, comprising 80 upregulated and 79 downregulated proteins (Figure 5C,D).

Cross-omics integration identified 16 key genes (KGs) that were consistently altered at both transcript and protein levels (Figure 5E). Co-expression analysis revealed strong associations among genes related to fatty acid metabolism and mitochondrial activity, such as *ACSF2*, *HSD17B10*, *EHHADH*, and *ECHDC1*, as well as between *CD74* and *MRPL41*, which are linked to immune function and ribosomal activity (Figure 5F). Functional annotation indicated that these KGs participate in pathways governing branched-chain amino acid catabolism, propanoate metabolism, and peroxisomal processes (Figure 5G,H). These results suggest that ovariectomy reshapes muscle metabolism by selectively modulating these pathways.

**Figure 6:**
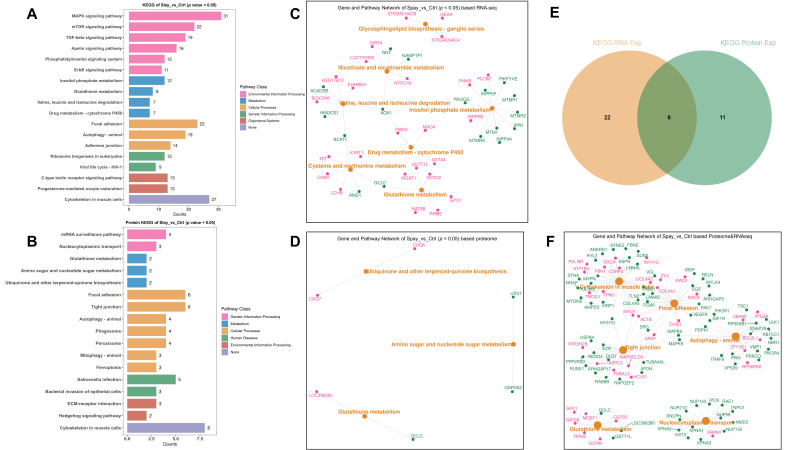
KEGG pathway annotation of transcriptomic and proteomic data from gastrocnemius muscle of Wuding hens at 160 days of age. (**A**) KEGG pathway enrichment of differentially expressed genes (DEGs) from transcriptome analysis. (**B**) KEGG pathway enrichment of differentially expressed proteins (DEPs) from proteome analysis. (**C**) Top enriched KEGG pathways from transcriptomic data along with associated gene sets. (**D**) Top enriched KEGG pathways from proteomic data along with corresponding gene sets. (**E**) KEGG pathways commonly enriched in both transcriptomic and proteomic datasets. (**F**) Representative gene sets enriched in the overlapping pathways; pink indicates upregulation and green indicates downregulation.

To further resolve pathway-level changes, we carried out KEGG enrichment analyses separately for DEGs and DEPs. Transcriptomic data highlighted significant enrichment in MAPK and mTOR signaling, as well as focal adhesion and cytoskeletal regulation (Figure 6A). Proteomic results showed a broadly consistent enrichment profile (Figure 6B), supporting coordinated changes at transcriptional and translational levels. Representative genes from these enriched pathways are illustrated in Figure 6C,D.

Six signaling pathways were found to be consistently enriched in both datasets (*p* < 0.05). These included focal adhesion, regulation of actin cytoskeleton, tight junction, autophagy, glutamate metabolism, and nucleocytoplasmic transport. Within these shared pathways, MAPK8, MAPK9, and ZYX emerged as central regulators (Figure 6E,F). Their involvement suggests that estrogen deficiency recruits stress signaling and structural remodeling networks to maintain muscle integrity during growth.

### 3.4. Effects of Ovariectomy and Growth Period on Lipid Metabolism in Wuding Hens

**Figure 7:**
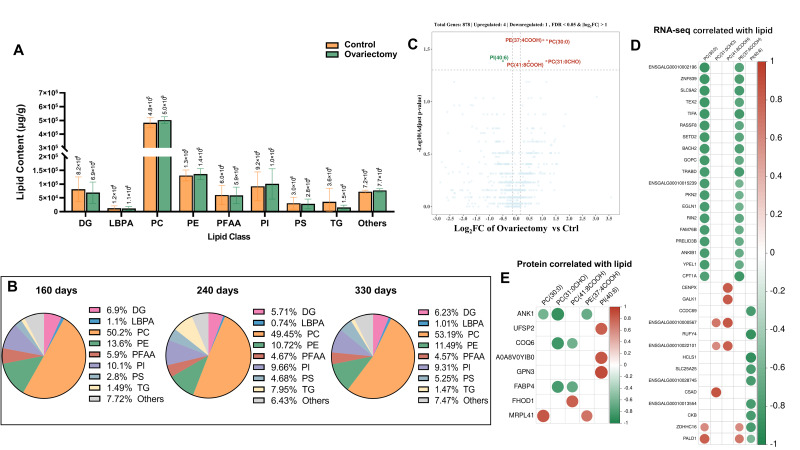
Lipid accumulation and integrative analyses in Wuding hens. (**A**) Comparison of total lipid content between ovariectomized and control groups. (**B**) Composition of lipid species in ovariectomized hens across three developmental stages. (**C**) Differential lipid accumulation between ovariectomized and control hens at 160 days of age. (**D**) Correlation analysis between differentially accumulated lipids and transcriptomic data. (**E**) Correlation analysis between differentially accumulated lipids and proteomic data.

Building on the transcriptomic and proteomic evidence of metabolic reprogramming after estrogen removal, we next examined lipid metabolism in skeletal muscle to determine whether similar changes occurred at the lipid level. We quantified total intramuscular fat and assessed lipid subclass composition in leg muscle from ovariectomized and intact hens at 160, 240, and 330 days of age. Total lipid content did not differ significantly between groups at any time point (Figure 7A), suggesting that ovariectomy does not alter the overall extent of fat deposition in muscle. However, differences emerged when lipid classes were analyzed. At 240 days, triglycerides (TG) accounted for 7.95% of the lipid pool in ovariectomized hens, compared with only 1.49% and 1.47% at 160 and 330 days, respectively (Figure 7B). This mid-growth increase indicates a temporary activation of lipogenic processes, possibly reflecting endocrine and metabolic shifts following ovarian hormone withdrawal.

To explore the molecular underpinnings of these changes, we conducted a combined lipidomic and transcriptomic analysis at 160 days. Four phospholipid species—PE (37:4COOH), PC (30:0), PC (31:0CHO), and PC (41:8COOH)—were significantly elevated in ovariectomized hens, whereas PI (40:6) was reduced (Figure 7C). These patterns indicate a selective remodeling of phospholipid metabolism in response to estrogen deficiency. Correlation analysis highlighted several notable gene–lipid associations. The accumulation of PC (30:0) and PE (37:4COOH) was positively correlated with *PALD1* and *ZDHHC16*, while *TEX2*, *TIFA*, and 17 additional genes showed strong negative correlations. PC (31:0CHO) abundance was positively linked to *ENSGALG00010000567*, *ENSGALG00010022101*, and *CSAD*, whereas PC (41:8COOH) was positively associated with these same genes plus *CENPX* and *GALK1* (Figure 7D). Conversely, PI (40:6) was negatively associated with nine genes, including *CCDC69*, *RUFY4*, and *HCLS1*, suggesting a role in phosphoinositide turnover or degradation.

Several candidate regulators also emerged with broader impacts on lipid remodeling. MRPL41 was positively correlated with PC (30:0) and PE (37:4COOH), whereas ANK1 showed the reverse; COQ6, FABP4, and ANK1 negatively correlated with PC (31:0CHO); FHOD1 was positively linked with PC (41:8COOH); and UFSP2, A0A8VOYIB0, and GPN3 showed positive correlations with PI (40:6) (Figure 7E). Many of these genes are known to participate in membrane remodeling, lipid oxidation, or intracellular transport, supporting their roles in maintaining lipid balance in the absence of estrogen.

## 4. Discussion

This study shows that surgical ovariectomy in Wuding hens markedly influences growth performance, carcass traits, muscle quality, and metabolic remodeling in skeletal muscle. By removing the ovaries, we created a physiologically relevant model of estrogen deficiency in female chickens. This model allowed us to probe how estrogen regulates somatic growth and muscle metabolism—an area that has received far less attention in females than in studies of male caponization.

One of the clearest findings was the extension of the growth period and the increase in final carcass yield in ovariectomized hens. At 330 days, body weight and carcass indices were significantly higher than in intact controls. This outcome can be explained by the suppression of estrogen-driven sexual maturation. In intact hens, a large proportion of nutrients and metabolic energy is diverted toward egg production once laying begins. Ovariectomy eliminates this reproductive investment, permitting continued allocation of resources to somatic growth. Similar results have been reported in pullets, where ovariectomy promoted body weight gain and carcass development compared to non-operated birds [13]. These findings parallel the well-known effects of caponization in males, which prolongs growth and increases carcass fat deposition [14,15,16]. Collectively, our data indicate that estrogen acts as a brake on somatic growth once reproduction begins, and its removal lifts this constraint, thereby extending the growth trajectory of hens.

Another notable outcome was the improvement in meat tenderness, especially in thigh muscles. Shear force was significantly reduced in ovariectomized hens at later stages, even though muscle fiber size and density did not differ from intact controls at 330 days. This is somewhat different from observations in younger ovariectomized hens, where smaller muscle fibers and higher intramuscular fat (IMF) content were reported, both of which correlated with tenderness [16]. In our long-term study, histological differences may have been masked by age-related hypertrophy, or thigh muscle may simply respond differently to estrogen removal compared with breast muscle. Nevertheless, the enhancement in tenderness appears to be linked to changes in lipid composition rather than fiber morphology. At 240 days, triglyceride (TG) proportion increased sharply in the ovariectomized group, suggesting a temporary shift toward marbling-type lipid deposition. Triglycerides can lubricate fibers and contribute to juiciness and tenderness. Similar improvements in sensory quality have been reported in both caponized roosters and ovariectomized hens, often associated with increased IMF or shifts in lipid partitioning rather than absolute fat content [16,17,18]. Comparable results have been reported in pigs, where castration-induced sex hormone deficiency led to elevated serum triglycerides and upregulated expression of lipid synthesis genes such as *FAS* and *ACC* [19], supporting the view that hormone deficiency promotes lipogenesis across species. In broilers, Sinanoglou et al. also demonstrated that caponization alters intramuscular lipid composition, increasing neutral lipid fractions and improving tenderness, which aligns with our findings in hens [17].

At the molecular level, ovariectomy induced distinct changes in signaling pathways central to muscle growth and adaptation. Transcriptomic and proteomic profiling showed consistent upregulation of MAPK8 and MAPK9, members of the stress-responsive JNK family. This pattern suggests enhanced activation of remodeling or inflammatory signaling. Estrogen normally dampens inflammatory responses and supports cytoskeletal stability, so its absence may increase reliance on MAPK signaling to maintain muscle structure under altered growth demands [14,20,21]. The concurrent upregulation of Zyxin (ZYX), a focal adhesion protein involved in cytoskeletal reorganization, reinforces this interpretation [22,23]. Comparable endocrine studies in mammals have shown that estrogen deficiency augments MAPK signaling activity while also affecting mitochondrial genes such as *COQ6*, leading to reduced oxidative capacity [24]. Similarly, ovariectomized chickens exhibit altered expression of lipid and mitochondrial genes (e.g., *LPL*, *PNPLA2*, *FABP4*), further confirming that estrogen withdrawal impacts both structural and metabolic gene networks [4].

Interestingly, components of the mTOR pathway were also affected. In mammals, estrogen deficiency is often associated with reduced Akt–mTOR activity and muscle atrophy [25]. Our data in hens, however, indicate a different trajectory: ovariectomized birds continued to grow, with no sign of wasting. This suggests either species-specific regulation of mTOR or the activation of compensatory anabolic mechanisms, possibly through growth hormone or IGF-1. The overlap between MAPK and mTOR signaling points to a complex network adjustment, where stress and growth signals are integrated differently in birds than in mammals. These findings emphasize that estrogen is not simply a reproductive hormone but also a key regulator of growth-related signaling in avian muscle.

Perhaps the most striking changes were observed in lipid metabolism. Although total IMF content did not differ between groups by 330 days, lipidomics revealed marked differences in composition. At mid-growth (240 days), ovariectomized hens showed a transient but significant increase in triglyceride proportion, along with selective changes in phospholipids. Estrogen has established roles in regulating lipid turnover and fat partitioning [26,27]. Its absence appears to promote neutral lipid storage, consistent with marbling, rather than altering overall fat content. This type of remodeling has also been described in caponized males, where improved tenderness and flavor occur without dramatic changes in total fat mass [16]. In pigs, castration has been shown to elevate serum cholesterol and triglycerides and upregulate lipogenic gene expression [19], whereas in chickens, ovariectomy induced significant transcriptomic changes in abdominal fat, including suppression of lipid breakdown genes and upregulation of steroid biosynthesis genes [4]. These parallels suggest that estrogen plays a conserved role in coordinating lipid storage and distribution across species.

Gene expression analyses provided further mechanistic insights. Upregulation of *FABP4*, a key fatty acid-binding protein, suggests enhanced lipid uptake and trafficking in muscle cells. Altered expression of *ANK1*, a cytoskeletal anchoring protein associated with tenderness and fat deposition traits in livestock [28,29], hints at changes in muscle membrane integrity that may facilitate fat incorporation. *COQ6*, involved in mitochondrial coenzyme Q synthesis and oxidative metabolism [30], was also modulated, consistent with shifts in muscle energy metabolism after estrogen removal. Our results are consistent with mammalian models, where estrogen deficiency upregulates *FABP4* in muscle tissue [31] and downregulates mitochondrial function genes such as *COQ6* [24]. This cross-species similarity strengthens the interpretation that estrogen is a central regulator of both lipid and energy metabolism in muscle.

Systemic effects were also evident in blood biochemistry. Serum total protein was higher in ovariectomized hens, particularly at early stages. In intact hens, large amounts of protein are directed toward yolk synthesis and egg production. In contrast, ovariectomized birds likely diverted protein toward somatic tissues and circulation. Estrogen influences hepatic protein synthesis [32,33,34]; its absence may therefore redistribute protein metabolism, enhancing growth and maintaining systemic balance. Comparable findings have been observed in roosters, where caponization elevated serum HDL, LDL, and total cholesterol compared with intact males [35]. In humans, postmenopausal women experience elevated triglyceride and LDL but reduced HDL due to estrogen decline [36], supporting the conclusion that estrogen withdrawal alters systemic protein and lipid metabolism across species.

Taken together, this study provides one of the most comprehensive analyses of estro-gen deficiency in female poultry. While male caponization has long been recognized as a tool for producing high-quality meat, comparable work in females has been scarce. Our results demonstrate that ovariectomy can prolong growth, improve carcass traits, and enhance tenderness, while reshaping lipid metabolism and signaling pathways. Although ovariectomy itself has little direct application in commercial production, the insights gained here have broader relevance. They not only highlight the role of estrogen in coordinating growth, lipid deposition, and muscle signaling, but also identify candidate targets—such as FABP4, MAPK8, and mTOR—for nutritional or pharmacological strategies aimed at improving meat quality.

## 5. Conclusions

In conclusion, ovariectomy in Wuding hens extended the growth period, increased carcass yield, improved thigh muscle tenderness, and selectively remodeled muscle lipid composition. Estrogen deficiency also altered MAPK and mTOR signaling networks and modulated expression of lipid- and energy-related genes such as *FABP4*, *ANK1*, and *COQ6*. These findings highlight estrogen’s broad role in skeletal muscle development and metabolism in hens and underscore its contribution to meat quality formation. While surgical ovariectomy is not a practical intervention for poultry production, the mechanistic insights gained here may inform alternative approaches to improving meat quality through hormonal, nutritional, or genetic strategies.

## Data Availability

The datasets generated and/or analyzed during the current study are available from the corresponding author on reasonable request.
